# Rectus Abdominis Fascia Plication Within DIEP Flap Breast Reconstruction: Impact on Abdominal Wall Morbidity and Patient Satisfaction Evaluated with the BREAST-Q

**DOI:** 10.3390/jcm14227939

**Published:** 2025-11-09

**Authors:** Beniamino Brunetti, Rosa Salzillo, Giuseppe A. G. Lombardo, Chiara Camilloni, Matteo Pazzaglia, Marco Morelli Coppola, Valeria Petrucci, Mauro Barone, Stefania Tenna, Paolo Persichetti

**Affiliations:** 1 Department of Plastic, Reconstructive and Aesthetic Surgery, Fondazione Policlinico Campus Bio-Medico di Roma, Via Alvaro del Portillo 200, 00128 Rome, Italy; b.brunetti@policlinicocampus.it (B.B.); camilloni.chiara@gmail.com (C.C.); matteo.pazzaglia@unicampus.it (M.P.); m.morellicoppola@policlinicocampus.it (M.M.C.); valeriapetruccimd@gmail.com (V.P.); maurosabbarone@gmail.com (M.B.); s.tenna@policlinicocampus.it (S.T.); p.persichetti@policlinicocampus.it (P.P.); 2 Burn & Plastic, Reconstructive and Aesthetic Surgery, Azienda Ospedaliera Cannizzaro, Via Messina 829, 95126 Catania, Italy; giuseppe.lombardo@unikore.it; 3 Department of Medicina e Chirurgia, Università degli Studi di Enna “Kore”, Piazza dell’Università, 94100 Enna, Italy

**Keywords:** autologous breast reconstruction, rectus abdominis diastasis, abdominal wall, microsurgery, DIEP flap, abdominoplasty, abdominal wall reconstruction, BREAST-Q

## Abstract

**Background**: The deep inferior epigastric perforator (DIEP) flap is the gold standard for autologous breast reconstruction, but donor site morbidity, such as abdominal bulging, remains a concern. Rectus fascia plication is a recognized treatment for rectus abdominis diastasis, yet its role in DIEP closure is scarcely evaluated. This study investigates whether plication reduces abdominal morbidity and improves patient satisfaction. **Methods:** A multicenter retrospective case–control study was performed on women who underwent unilateral DIEP breast reconstruction between 2018 and 2024. Patients were allocated to the plication or control group according to operative notes. Outcomes included abdominal complications, postoperative bulging (clinically and photographically assessed), and patient-reported satisfaction using the BREAST-Q. Standardized postoperative photographs were also rated by blinded expert surgeons. **Results**: Sixty-two patients met the inclusion criteria: 25 with fascia plication and 37 without. Groups were comparable in demographics and surgical details, though diastasis was more prevalent in the plication group (4.1 cm vs. 0 cm, *p* < 0.0001). Plication did not increase early or late complications. Abdominal bulge occurred in 16.2% of controls and 0% of the plication group (*p* = 0.0727). Logistic regression confirmed a significant protective effect (OR = 0.095, 95% CI 0.001–0.87, *p* = 0.034). BREAST-Q “Physical well-being: abdomen” scores were higher with plication (35.7 ± 18.0 vs. 23.0 ± 17.2, *p* = 0.0316), which was confirmed by multivariate analysis. Expert photographic assessments showed no significant differences. **Conclusions**: Rectus fascia plication during DIEP flap breast reconstruction is safe, improves abdominal well-being, and reduces postoperative bulging, especially in patients with preoperative diastasis. This additional step may represent a simple and effective strategy to enhance donor site outcomes.

## 1. Introduction

Breast cancer remains the most prevalent malignancy among adult women worldwide, with approximately one in eight women being affected during their lifetime [1]. Although conservative surgical approaches such as quadrantectomy and oncoplastic techniques are increasingly employed, mastectomy continues to be an essential treatment in many clinical scenarios. Advances in reconstructive surgery have allowed for the restoration of breast shape and volume using autologous tissue, significantly improving psychological well-being and overall quality of life in affected patients. Amongst available options, the deep inferior epigastric perforator (DIEP) flap has emerged as the gold standard for autologous breast reconstruction due to its ability to provide natural contour and texture while preserving abdominal muscle integrity [2].

Concurrently, rectus diastasis, a condition characterized by the separation of the rectus abdominis muscles, affects up to 30% of women following pregnancy or significant weight fluctuations [3]. This condition can result in a range of functional and aesthetic issues, including abdominal bulging, core instability, back pain, digestive discomfort, and urinary incontinence. Treatment is surgical correction through fascial plication, with the aim of restoring abdominal wall integrity and improving quality of life [4].

The DIEP flap procedure provides a unique opportunity to simultaneously address breast reconstruction and improve abdominal contour. Despite the advances in perforator dissection allowing for preservation of rectus abdominis muscles and their motor branches, abdominal morbidity due to nerve section and fascial weakening still occurs, leading to rare but troublesome complications such as postoperative hernia or, more frequently, to the development of clinically visible or sub-clinical abdominal bulge. In this scenario, abdominal fascia plication could play a role in limiting or preventing the occurrence of such complications. Nevertheless, the impact of concurrent rectus fascia plication on abdominal morbidity and patient satisfaction within DIEP flap breast reconstruction procedures remains scarcely assessed. While some surgeons routinely incorporate plication to enhance abdominal contour and mitigate postoperative bulging, concerns persist regarding potential complications or unnecessary procedures in patients without pre-existing diastasis.

In recent years, the integration of patient-reported outcome measures (PROMs) into surgical research has gained increasing relevance: the BREAST-Q, validated in Italian, allows for the standardized evaluation of patient satisfaction and quality of life following breast reconstruction, offering valuable insights beyond traditional clinical endpoints.

This study aims to evaluate the abdominal outcomes and patient satisfaction in women undergoing DIEP flap breast reconstruction, comparing those who received rectus fascia plication to those who did not. By assessing clinical findings, PROMs, and blinded expert evaluations of postoperative photographs, this study seeks to clarify whether fascial plication should be routinely considered during DIEP flap breast reconstruction to optimize both functional and aesthetic results.

## 2. Materials and Methods

This is a multicenter retrospective case–control study carried out between the Plastic Surgery Department of Campus Bio-Medico University Hospital in Rome and the Burn and Plastic, Reconstructive and Aesthetic Surgery at Azienda Ospedaliera Cannizzaro of Catania, Italy. The study was carried out according to the Helsinki Declaration.

The electronic medical record system of both institutions was searched for DIEP autologous breast reconstruction performed between 2018 and 2024. Data collection was carried out by two independent researchers at each institution, who later went over and reconciled their data. Inclusion criteria were as follows: female patients, age > 18 years, diagnosis of breast cancer treated with mastectomy, immediate or delayed unilateral autologous reconstruction with DIEP flap, follow-up of at least 6 months, signed informed consent, and good proficiency in the Italian language.

Exclusion criteria were as follows: bilateral DIEP flap harvesting for bilateral or unilateral stacked microsurgical breast reconstruction, microsurgical breast reconstruction with flaps alternative to single-pedicle DIEP (PAP—profunda femoris artery perforator flap, and TRAM—transverse rectus myocutaneous flap), autologous reconstruction with locoregional flaps (latissimus dorsi [5]), implant breast reconstruction, hybrid breast reconstruction, previous abdominal surgery via laparotomic access, previous abdominoplasty, prior diagnosis of connective tissue disorders such as Marfan or Ehlers–Danlos syndrome, intraoperative abdominal wall repair using techniques different from direct fascial suture (e.g., use of biological or synthetic meshes), rectus abdominis plication performed as a delayed secondary procedure, insufficient understanding of the Italian language by the patient, lack of informed consent, and patients lost to follow-up.

Patients were assigned to the study group if a rectus abdominis plication was performed during DIEP flap donor site closure (as written in the OP note); otherwise, they were assigned to the control group. Until 2021, plication was performed only for patients with significant intraoperative diastasis or laxity. After 2021, it was then offered to all patients with even minimal diastasis, or in the absence of diastasis, to improve aesthetic outcomes.

All flaps included in the study were true DIEP flaps, meaning that no muscle was harvested together with the perforator(s) (or a minimal amount was included, when some muscle fibers could not be safely dissected away from the perforator). A muscle-sparing procedure was attempted in all cases, and the only sacrificed nerves were those adherent to the perforator(s). In these few cases, the nerve was not repaired since, according to the literature, the sacrifice of small nerves is not linked to worse outcomes [6].

Management of the abdominal wall donor site differed between the two groups. While the rectus abdominis muscle fasciotomy was similarly closed with absorbable sutures (Vicryl 2/0), midline rectus abdominis fascia plication was performed only in the study group, plicating a vertical ellipse of fascia extending from the xyphoid process to the pubis with non-absorbable single interrupted stitches (Prolene 0). The technique of abdominal wall closure did not differ between the two centers involved in the study.

Extracted data were as follows: Patient age at the time of surgery, comorbidities, intraoperative timing (operative time, ischemia time), early complications (i.e., occurring within 5–7 days after surgery), late complications (within 3 months after surgery), and the number and type of reoperations involving the abdominal region. Diastasis width was measured according to the preoperative CT scan. Among the data, flap weight was not recorded due to the vast majority of procedures being delayed ones, rendering an exact match with mastectomy weight impossible; an aesthetic match to the contralateral breast was carried out instead.

The postoperative ambulation schedule was similar between the two groups. Pain control data were not collected, which represents a study limitation; however, pain was generally comparable between groups, as all patients received a TAP (transverse abdominal plane) block. Follow-up was carried out as an outpatient visit at least 6 months after the DIEP procedure. The follow-up program was similar for all patients. They were instructed to wear compression garments for 2–3 months, start aerobic exercise after 2 months, and begin muscle-strengthening exercises from 6 months postoperatively.

Bulging was defined, according to the literature, as any postoperative protrusion of the abdominal wall in a standing position, either clinically evident to the examining surgeon or reported by the patient. Bulging was distinguished from a hernia by the absence of a fascial defect and lack of a hernia sac. Patients were clinically evaluated for abdominal bulging in standing and lying positions, both at rest and while performing a Valsalva maneuver. Bulging cases were also examined by analyzing standardized postoperative photographs (compared to preoperative images) in frontal, oblique, and lateral projections, as well as during the diving maneuver. The differential diagnosis between bulging and herniation was performed with ultrasound, which demonstrated fascial continuity and muscular thinning in bulging.

Patient satisfaction was assessed by administering a validated Italian-language version of the BREAST-Q (reconstruction module) questionnaire at least 6 months after surgery. Data from both groups were compared to identify the most effective approach for addressing abdominal fascial laxity and rectus abdominis diastasis, specifically to determine whether it is advantageous to correct this condition.

Furthermore, standardized postoperative photographs of patients in each group were evaluated by two expert plastic surgeons who specialized in autologous breast reconstruction, and who were external to the study and blinded to its objectives and methodology, in order to objectively assess the aesthetic outcomes of the abdomen with and without diastasis correction (single-blind design). Evaluation was conducted using a 5-point Likert scale, where 1 represented the poorest outcome and 5 represented the best.

### Statistical Analysis

Normality of the study sample was checked using a Shapiro–Wilk test. Variables following a Gaussian distribution in the two groups were compared using a Student’s t test with 95% confidence intervals and two-tailed *p* values, whereas variables not following a Gaussian distribution in the two groups were compared using a Mann–Whitney U test with 95% confidence intervals and two-tailed *p* values. Nominal variables were analyzed using Fisher’s exact test with two-tailed *p* values. A multivariable linear regression model was fitted with “Physical well-being: abdomen” as the dependent variable and diastasis, BMI, and abdominal bulging as predictors. Model assumptions (normality and homoscedasticity of residuals) were checked, and a log-transformation of the dependent variable was applied to meet these assumptions. Abdominal bulges’ correlation with diastasis correction was examined via a penalized logistic regression. Spearman’s rank correlation was used to check the correlation between Likert scale evaluations. R and RStudio (version 4.3.2, R Foundation for Statistical Computing, Vienna, Austria) were used for statistical analysis. Statistical significance was defined as *p* < 0.05 (*).

## 3. Results

A total of 62 patients met the inclusion criteria: 37 patients were enrolled in the control group (no plication) and 25 in the plication group. Demographic and surgical characteristics were similar between groups, with no statistically significant differences in age (52.2 ± 9.0 vs. 50.9 ± 9.9, *p* = 0.618), BMI (25.7 ± 5.5 vs. 23.9 ± 5.6, *p* = 0.130), smoking (13.5% vs. 19.0%, *p* > 0.999), previous pregnancies (94.6% vs. 96.0%, *p* > 0.999), previous miscarriages (21.6% vs. 20.0%, *p* > 0.999), previous radiotherapy (70.3% vs. 76.0%, *p* = 0.774), or surgical timing (in the control group, 5.4% of breast reconstructions were immediate, versus 16% in the study group, *p* = 0.210).

There were also no significant differences in intraoperative details such as perforator choice (86.5% of flaps were based on the medial row in the control group versus 84.0% in the study group, *p* > 0.999) or insetting type (73% was external in the control group versus 64% in the study group, *p* = 0.576) (Table 1).

Preoperative diastasis was significantly more prevalent in the plication group (mean: 4.1 cm, SD: 1.7) compared to the control group (mean: 2.7 cm, SD: 1.3, *p* = 0.0012).

There were no significant differences in early or late postoperative complications between groups. Notably, compared to the control group, the plication group did not experience increased rates of infection (16.2% vs. 4.0%, *p* = 0.225), abdominal wound dehiscence (18.9% vs. 8.0%, *p* = 0.292), partial flap loss (8.1% vs. 8.0%, *p* > 0.999), or liponecrosis (8.1% vs. 4.0%, *p* = 0.642).

Total flap loss occurred in one patient (4%) in the plication group and in none from the control group, without statistical significance (*p* = 0.403).

**Figure 1:**
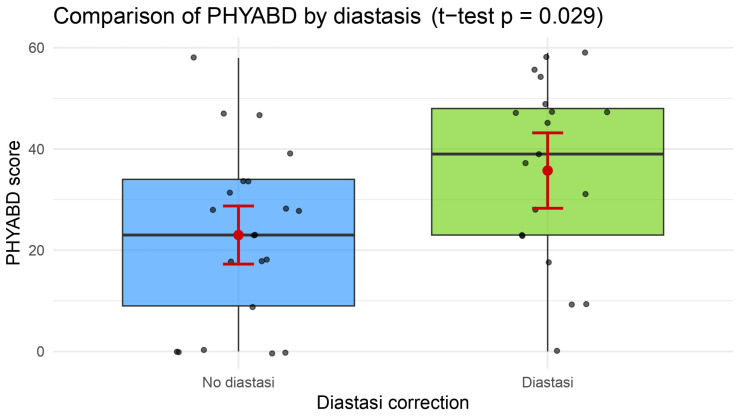
A box plot with the results of comparative tests on the “Physical well-being: abdomen” scale, showing a statistically significant higher score in the study group (see also Table 2).

**Figure 2:**
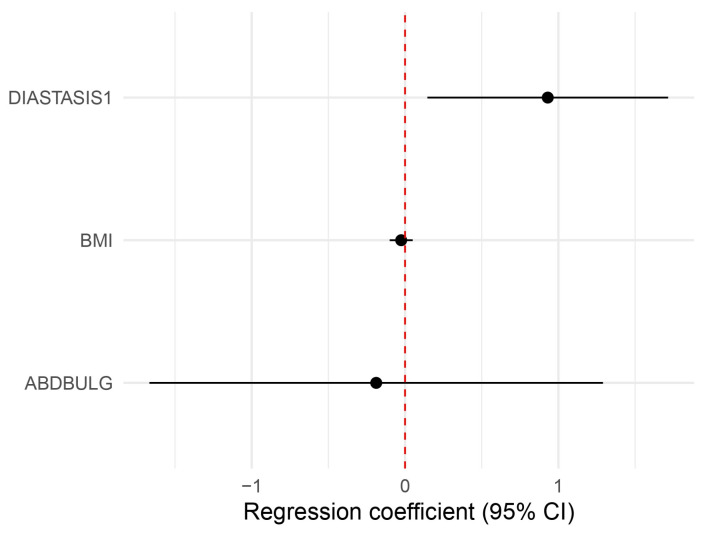
A forest plot of the multivariate analysis that shows a statistically significant influence of diastasis plication on the “Physical well-being: abdomen” scale, even when controlling for potentially confounding factors like BMI and bulging (see also Table 3).

Patient-reported outcomes collected via the BREAST-Q showed a higher score in the “Physical well-being: abdomen” scale for the plication group (mean: 35.7 ± 18.0 vs. 23.0 ± 17.2, *p* = 0.0316) (Table 2, Figure 1). This result was confirmed through a multivariate analysis, where diastasis plication confirmed its role in elevating patient satisfaction, even when controlling for BMI and postoperative abdominal bulge (*p* = 0.026, CI 1.16–5–56, β = 2.54) (Table 3, Figure 2).

**Figure 3:**
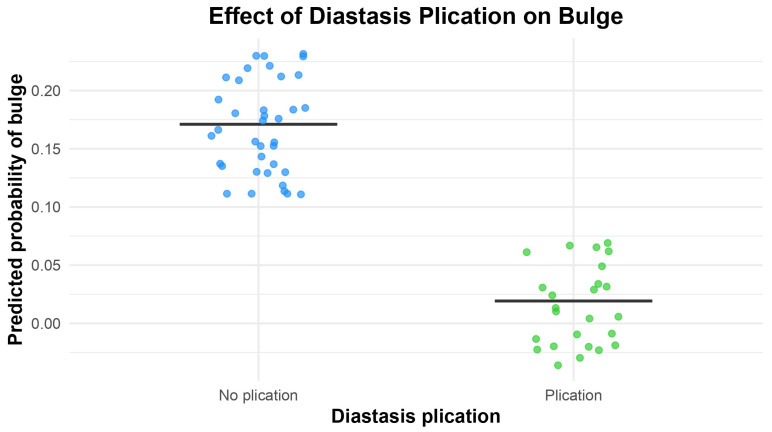
A box plot of the results of the penalized Firth regression performed with postoperative abdominal bulging as the independent variable, showing a statistically significant protective influence of diastasis plication for preventing it (also see Table 3).

Postoperative abdominal bulging occurred in six patients in total (9.7%): six patients (16.2%) in the control group and zero patients in the plication group, with a clear trend toward a statistically significant difference (*p* = 0.0727) at comparative tests. Bulging also appeared not to be linked to medial or lateral row dissection (*p* = 0.206, with a small trend toward a higher risk linked to lateral row dissection) and neither to the number of perforators included, i.e., one or more than one (*p* = 0.999). When more than one perforator was dissected, they always belonged to the same row, whether medial or lateral. The association between the occurrence of abdominal bulge and diastasis correction was also evaluated via logistic regression, controlled for confounding factors like the number of perforators and perforator row. Here, patients who underwent diastasis plication had a substantially lower risk of developing abdominal bulge compared to those who did not (OR = 0.092, 95% CI: 0.001–0.83, *p* = 0.031), meaning that diastasis plication reduces the odds of developing a clinically evident abdominal bulge by approximately 95% compared to no plication (Table 3, Figure 3).

Satisfaction with the surgeon was high in both groups and did not differ significantly (Table 4). As for the expert surgeons’ satisfaction analysis, no statistically significant difference was found between the control and the study group (3.64 ± 0.83 vs. 3.56 ± 0.96, *p* = 0.550). Spearman’s rank correlation showed a good correlation between the two surgeons’ opinions (ρ = 0.716, *p* < 0.0001).

## 4. Discussion

The DIEP flap has established itself as the gold standard in autologous breast reconstruction, offering not only reliable vascular anatomy and preservation of abdominal wall musculature, but also an intrinsic aesthetic benefit at the donor site. In many patients, the resulting abdominal contour resembles that of a cosmetic abdominoplasty, which contributes positively to postoperative body image and overall satisfaction.

The extensive literature addresses donor site complications in abdominal flaps for breast reconstruction [7,8,9], methods for the safeguarding of donor site anatomy during dissection (such as the robot-assisted harvest [10], or the medial paramuscular approach to perforator dissection [11]), placing a mesh to reinforce the abdominal wall [12], and better wound healing by using NPWT [13]. Several authors have compared the conventional approach to minimally invasive ones, with varying results [14,15]. Factors influencing patients’ satisfaction have also been analyzed in the literature [16], as well as the effects on donor site functionality of both abdominal flaps [17] and of other flaps employed in breast reconstruction [18]. On the other hand, the role of rectus diastasis plication in DIEP flap patients has remained largely unexplored: while plication is a well-established surgical approach to correct abdominal wall laxity, its systematic application during DIEP flap closure is still debated. This is particularly relevant since diastasis recti is a very common and often underdiagnosed abnormality, and many breast cancer patients undergoing DIEP reconstruction may already present with varying degrees of rectus diastasis due to previous pregnancies and/or weight loss, which, being associated with nerve section or fascial weakening, may lead to the development of donor site complications.

Rates of abdominal wall morbidity (bulging and hernia) reported in the literature gravitate around 10%, with bulging being much more common than hernia [19,20].

Our retrospective multicenter case–control study is, to our knowledge, the first to systematically evaluate the impact of concurrent rectus fascia plication in this patient population. By comparing patients who underwent fascial plication during DIEP donor site closure to those who did not, and combining both objective clinical assessment and patient-reported outcome measures (PROMs), we aimed to provide a comprehensive evaluation of outcomes.

**Figure 4:**
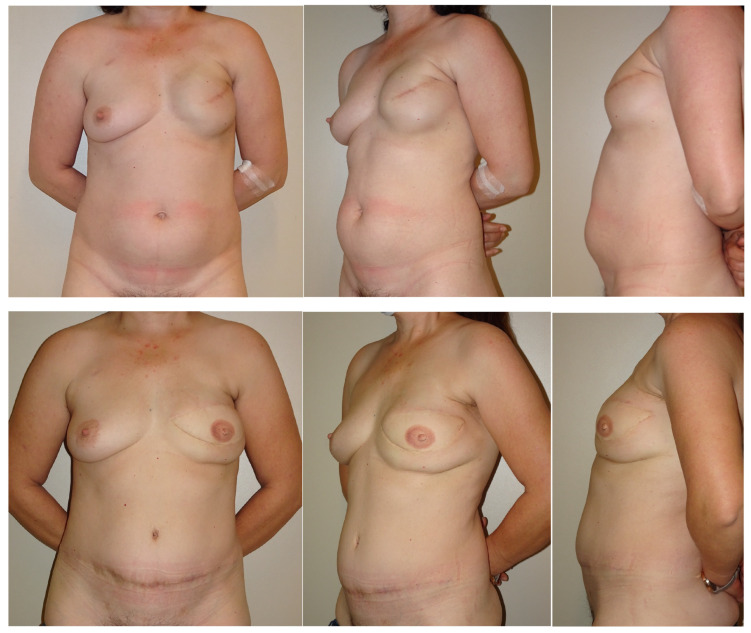

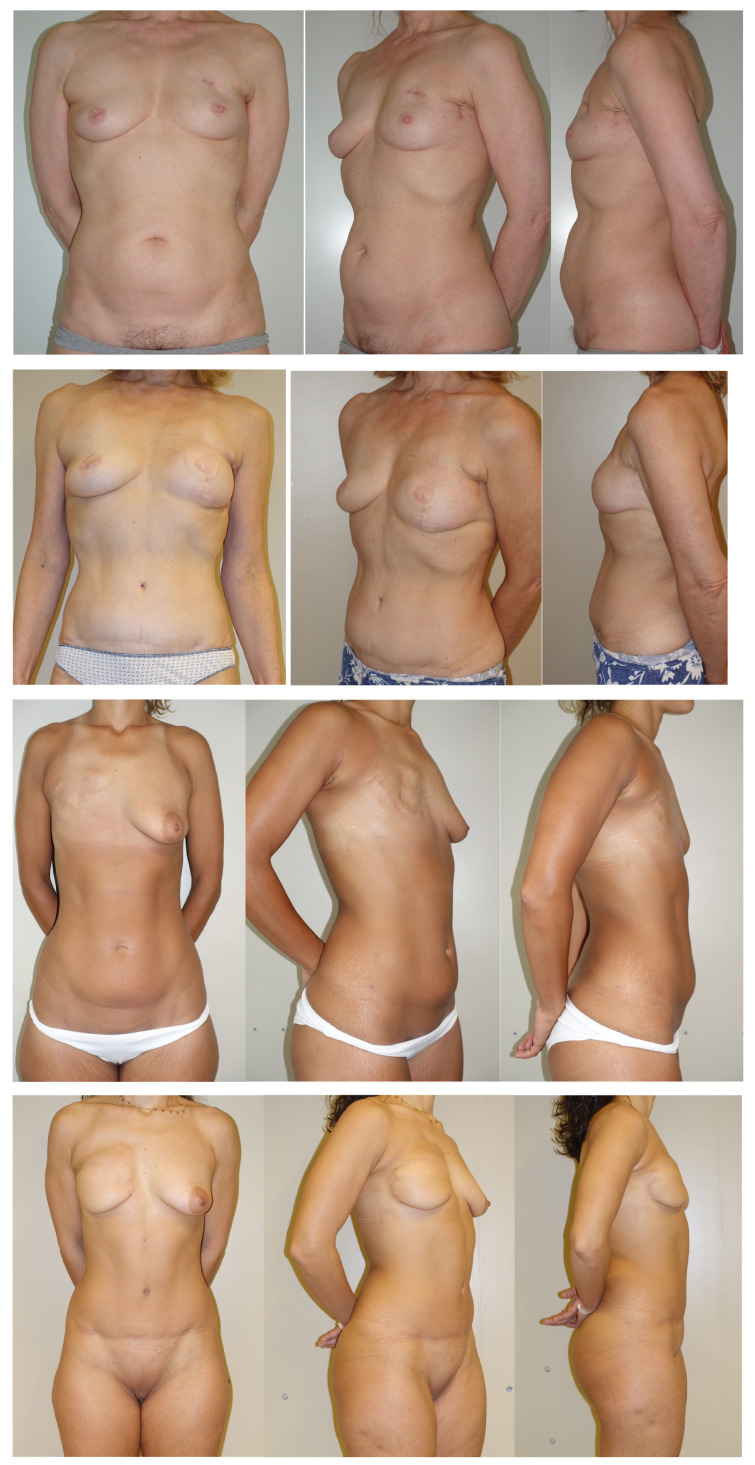
Comparison between the abdominal contour in a patient who did not undergo diastasis plication (control group) versus one who did (study group). First row: Control patient, pre-op pictures. Second row: Control patient, post-op pictures. Notice the abdominal bulging still present. Third row: Study patient 1, preop pictures. Fourth row: Study patient 1, post-op pictures. Notice the improved abdominal contour. Sixth row: Study patient 3, post-op pictures. Notice the improved abdominal contour. The patient is now scheduled for NAC (nipple–areola complex) reconstruction and breast simmetrization.

Patients who underwent plication for preoperative diastasis demonstrated better satisfaction with the donor sites’ physical well-being and cosmetics, without any increase in surgical morbidity (Figure 4). These improvements may reflect enhanced core stability and body image associated with the correction of abdominal laxity. Although our bulge incidence may appear higher than expected, this data may simply be explained by the small sample size and, furthermore, a lower incidence of postoperative abdominal bulging in the plication group was observed: this strongly suggests that abdominal fascia plication may have a prophylactic role in preventing postoperative clinically evident abdominal bulging. Both of these findings support the potential clinical utility of rectus fascia plication during DIEP flap breast reconstruction in appropriately selected patients.

Importantly, fascial plication did not result in increased operative risks. Complication rates remained low and comparable to the control group, suggesting this additional maneuver can be safely carried out during donor site closure.

Moreover, blinded expert evaluation of standardized postoperative photographs allowed for an objective assessment of abdominal contour. No significance or trend was observed; however, this may simply be explained as a consequence of the small sample size.

The primary limitation of this study lies in its limited sample size, particularly in the plication group, which reduced the power to detect statistically significant differences. Furthermore, the retrospective design inherently carries risks of selection bias, and although data collection was standardized and involved independent reviewers, unmeasured confounders may still have influence outcomes. The exclusion of patients who received rectus abdominis plication as a secondary delayed procedure is also a limitation. Our data confirms that fascial plication was selectively performed in patients with clinically evident abdominal wall laxity. This might introduce a selection bias in the study, since patients with a clinically evident or intra-operatively evident diastasis are more likely to have their diastasis recorded and receive the additional plication procedure, whereas patients without or with minimal (less than 2 cm) diastasis were less likely to have it corrected.

Additionally, while the multicenter nature adds generalizability, differences in surgical technique and patient demographics across the two centers may introduce variability. However, the consistent directional trends observed in both clinical and PROM data suggest that plication may offer real-world benefits worthy of further investigation.

Future prospective studies with larger cohorts and standardized assessment of diastasis severity are warranted to confirm these preliminary findings and define clear indications for fascial plication during DIEP flap donor site closure.

Despite these limitations, our findings support the safety and effectiveness of routine fascial plication during DIEP flap donor site closure. Based on our experience, we now routinely assess for preoperative rectus diastasis and perform plication not only in patients with clinically evident diastasis, but, in general, in all patients to prevent the development of clinically evident abdominal bulge and optimize both functional and aesthetic outcomes.

## 5. Conclusions

Rectus fascia plication during DIEP flap breast reconstruction improves patient satisfaction and may have a role in reducing postoperative abdominal bulging rates when performed in patients with preoperative diastasis undergoing DIEP flap breast reconstruction. The procedure appears safe and offers a simple yet effective means to enhance donor site aesthetics and function.

## Figures and Tables

**Table 1:** Results of comparative tests regarding patients’ demographic data, history, procedure details, and postoperative complications in the control group versus the study group.

	Group 1, Value *n* = 37	Group 2, Value *n* = 25	*p* Value
Age	52.2± 9.0	50.9 ± 9.9	0.6181
BMI	25.7 ± 5.5	23.9 ± 5.6	0.1302
Smoking	13.5%	16.0%	>0.9999
Previous pregnancies	94.6%	96.0%	>0.9999
Previous miscarriages	21.6%	20.0%	>0.9999
Previous RT	70.3%	76.0%	0.7736
Timing: delayed	94.6%	84.0%	0.2097
Perforator row: medial	86.5%	84.0%	>0.9999
More than one perforator included (always same row)	35.1%	32.0%	>0.9999
Insetting: external	73.0%	64.0%	0.5761
Insetting: buried	27.0%	36.0%	0.5761
Preoperative diastasis (cm)	2.7 ± 1.3	4.1 ± 1.7	0.0012 *
Abdominal bulge	16.2%	0%	0.07268
Total flap loss	0%	4.2%	0.4032
Partial flap loss	8.1%	8.0%	>0.9999
Liponecrosis	8.1%	4.2%	0.6418
Abdominal dehiscence	18.9%	8.0%	0.2916
Infection	16.2%	4.2%	0.2254

* *p* > 0.05, statistically significant.

**Table 2:** Results of the BREAST-Q (reconstructive module) scales in the control group versus the study group.

	Group 1 Value	Group 2 Value	*p* Value
Psychosocial well-being	63.0 ± 24.3	61.6 ± 18.9	0.8354
Sexual well-being	55.2 ± 20.3	49.8 ± 24.4	0.4520
Satisfaction with breast	58.6 ± 21.9	57.1 ± 13.7	0.7803
Physical well-being: chest	36.3 ± 26.8	32.5 ± 22.0	0.7332
Physical well-being: abdomen	23.0 ± 17.2	35.7 ± 18.0	0.0316 *
Satisfaction with surgeon	90.2± 14.6	94.4 ± 8.8	0.5124

* *p* > 0.05, statistically significant.

**Table 3:** Results of multivariate analysis (top) regarding the “Physical well-being: abdomen” BREAST-Q scale, showing a correlation with diastasis plication, and results of logistical regression (bottom) regarding postoperative clinically evident abdominal bulging, which showcases diastasis plication as a protective factor against its development.* *p* > 0.05, statistically significant.

**Physical Well-Being: Abdomen**	***p***	**CI**	**Beta Coef**
Diastasis plication	0.02609	1.1566914 5.558957	2.5357440 *
BMI	0.51578	0.9045022 1.051272	0.9751298
Abdominal Bulge	0.80461	0.1888424 3.635329	0.8285556
**Abdominal Bulge**	***p***	**CI**	**OR**
Diastasis plication	0.03121	0.0006948 0.8388511	0.0921519 *
Number of perforators	0.86855	0.1770843 6.5591235	1.1578344
Lateral row	0.13784	0.5982644 29.481968	4.3247184

**Table 4:** Expert surgeons’ evaluation results.* *p* > 0.05, statistically significant.

	**Surgeon 1, Mean ± SD**	**Surgeon 2, Mean ± SD**	***p* Value**
Group 1	3.93 ± 0.91	3.35 ± 0.90	0.112
Group 2	3.50 ± 1.10	3.62 ± 0.92	0.507
	**Control Group**	**Study Group**	***p* Value**
Surgeon global score	3.64 ± 0.83	3.56 ± 0.96	0.550
Spearman’s rank correlation	Rho = 0.716		*p* < 0.0001 *

## Data Availability

Data available on request from the authors.

## Abbreviations

The following abbreviations are used in this manuscript:

DIEP	Deep inferior epigastric artery perforator
PAP	Profunda artery perforator
TRAM	Transverse rectus abdominis myocutaneous
